# Bibliography

**Published:** 1904-11

**Authors:** 


### Bibliography.

A Text-Book of Dental Pathology and Therapeutics for Students and Practitioners. By Henry H. Burchard, M.D., D.D.S., late Special Lecturer on Dental Pathology and Therapeutics in the Philadelphia Dental College. Revised by Otto E. Inglis, D.D.S., Professor of Dental Pathology and Therapeutics in the Philadelphia Dental College. Second Edition. Illustrated with five hundred and forty-five Engravings and a Colored Plate. Lea Brothers & Co., Philadelphia and New York, 1904.

It is six years since (1898) the long-to-be-lamented Burchard wrote the preface to the first edition of this book. No attempt has been made since to reproduce it under proper editorial revision until the present time. The difficulties attending this were well understood by those conversant with the book as originally prepared. It was well understood that much of it would necessarily have to be rewritten. Professor Inglis undertook this serious task, and has completed it in a volume of six hundred and thirty-nine pages, against five hundred and eighty-five in the first edition. This, however, does not indicate the difference in pages between the two volumes, as forty-three pages have been eliminated by omitting the section on Pharmacology in the first edition.

The editor has followed Dr. Burchard in the arrangement of chapters, but has so thoroughly revised the text that the book must be reviewed, in its second edition, as an original production, the editor standing sponsor for all that has been written in both editions. There can be but one opinion, it is presumed, as to the faithfulness of the editor to his own convictions while, at the same time, retaining an almost sacred devotion to the man who originally planned and wrote the book. That he has succeeded in everything, as some may have desired, is not to be expected, but that he has brought the work up to present standards must be conceded. Whatever criticisms the reviewer may feel called upon to make after a careful perusal of its contents are simply the difference of individuals and methods of teaching, and may not be regarded as directly detracting from the value of the book as a whole.

The chapter on Inflammation is an improvement on that by Burchard, but it does not seem to the reviewer to be a full statement of this very important foundation subject. The reviewer recognizes the value and necessity for condensation in a book of this character, but condensation can be secured without omitting essentials, and these seem to have been to some extent excluded. The history of the work of histologists that led up to the final rediscovery of Conheim, from Döllinger (1819), through Williams, Waller, Von Recklinghausen, Beale, and others, would not have occupied much room, and would have been not only an instructive lesson to students, but would have given credit justly due to these pioneers in histological discovery. Further, the chapter fails to do justice to the classical terms of Redness, Heat, Swelling, Pain, and Impaired Function. These all cover very important facts, and are as important to-day in modern study of abnormal conditions as they were at any former period.

Necrosis of the jaw is very inadequately treated from the standpoint of the reviewer. The dentist should regard this as one of the important pathological problems to be met and conquered, and it will not do to relegate it entirely to the oral surgeon. Hence, while it may at the last fall into the hands of the specialist, its preliminary treatment should be, as a rule, in those of the dentist. Aside from this, the ever-present danger of producing serious lesions through modern methods of force in operating, and resulting in necrotic conditions, seems to make more extended explanation absolutely necessary. No allusion is made to these traumatic disturbances or to the means of avoiding or treating them.

A typographical error occurs on page 141, where Gyse's name is spelled Geise.

The chapter on Dentition is fairly well treated. The editor adopts Constant's suggestion as to the causes producing eruption of the teeth. It is, of course, a matter of theory and not one of proof, hence it is hardly worth while to devote much attention to it, but the illustration of the truth of Constant's theory does not seem very conclusive, for he says, "A simple accident demonstrated this to the editor. While excavating with a large bur the softened dentine about a decayed pulp-chamber, the cementum was widely removed from the pericemental tissue beneath, which latter fortunately remained unbroken. It immediately protruded into the perforation." This the editor regards as the result of internal pressure. It is questionable whether this furnishes proof of a pressure sufficient to force a tooth towards the gum, producing resorption of that and the enveloping process.

The editor is at times profuse in his credits, and at others, as already stated, he seems to have failed to do justice. This is marked wherever it becomes necessary to make use of the reviewer's name and work. On page 201, in writing on "Pathological Second Dentition," he explains that "The lower second molars may cause some irritation owing to an insufficient development of the jaw at the angle, leaving an inadequate accommodation for the crown. At about nine years of age the second molar occupies the angle of the jaw in much the same position as shown in Fig. 101 for the third molar. If held back, a pathological condition equivalent to that occurring in the temporary teeth may result. . . . Truman has prevented a threatened second attack of this sort by deep incisions in the gum over the site of the crown." This is all very imperfectly stated, which is all the more strange, as the editor quotes the facts from an article written by the reviewer in 1899 and read before the National Dental Association. The entire subject-matter, from the time the second molar of the mandible passed from the ramus to assume the direct vertical, was based on original investigation by the reviewer some twenty years previous to that time, and these observations had been so fully confirmed by experience that he had felt justified in calling attention to it as an unobserved pathological problem in second dentition. If any one had noticed this and its resultant nervous reflexes, it had not been observed in the reviewer's reading.

The subject of impacted third molars is generally satisfactory. The editor follows Cryer mainly both in illustration and general ideas. Much might be said of value to the student, but is not mentioned here, especially as to the possible pathological conditions following malposition, where the third molar of the lower jaw abuts against the second molar in an inclined position, inviting caries and final exposure of the pulp.

On page 256 the editor is in error in stating that it was first suggested by "Truman that erosion is due to an altered secretion of the mucus-forming glands of the lip which lie in close relation to them. Truman found these to secrete an acid at night, while during the day the secretion might be alkaline." It may be added to this that Truman found nothing of the kind, but what he did find, after an extended investigation as to the cause of erosion and abrasion, was that at night, when the alkaline saliva practically ceased to flow from the salivary glands, the fermentative process was exceedingly active, with the result that tests at night and early in the morning gave invariably an acid reaction. The lip being held directly upon the anterior teeth during this period acted as an agent to retain the acid fluids in contact with the enamel, and the mucous membranes of the cheeks did the same service for the buccal surfaces of the posterior teeth. Kirk was the one who demonstrated that the mucus glands of the lip gave an acid reaction during the day. His tests were not carried into the night, but it was presumed that there was a continuous action during the twenty-four hours more pronounced during the periods of rest at night.

The chapter on "Stains of the Enamel and Dentine" is satisfactory although brief, but the treatment will not be of much use to the average student. There is great need of more practical detail on this very important branch of operative procedures.

The history of dental caries as given under that chapter is lacking in much that would have been of value. While Miller completed the labor of investigation of dental caries, his work supplemented that of a long line of investigators, but the editor begins his history of the micro-organic theory with Leber and Rottenstein, whereas it should have gone back to Erdl, Ficinus, and Klencke. Writers of books are apt to forget that they are making a repository of history, and that this requires careful compilation of facts, that future writers may not be led into error.

The following very curious statement, to be found on page 327, seems to require notice. He states that "Dentine cannot become inflamed, as leucocytes cannot enter the tubules; nevertheless the irritability of the fibrillæ, like that of other protoplasm, may be heightened." It is altogether a novel idea that leucocytes by entering the tubuli could arouse inflammation. Have blood-vessels ever been discovered permeating dentine? and are the leucocytes the originators of inflamed areas, or, are they simply the final products, through an abnormal condition in the circulation? This certainly should be corrected in future editions, as it fails to properly explain the subject.

"Hypersensitive Dentine and Therapeutics" is well treated from the therapeutical side of the question, but is not equally as well considered from the histological. To mention hypersensitive dentine carries the thought at once back to Tomes, but his name is not mentioned in connection with the discovery of the fibrillæ that bear his name.

This neglect in giving proper credit is manifest in the use of silver nitrate on children's teeth. This properly belongs to the late Dr. Stebbins, and its importance in controlling caries in deciduous teeth should have decided the editor in giving a very full report of his procedures and the results obtained.

The following paragraph is very far from the position held by some, at least, of the leading histological observers of to-day. The editor states, on page 355, "That the caries fungi may be present in the mouth and be harmless, unless conditions favor the formation of gelatinous plaques upon the teeth, as has been shown by Black and Williams.

"These facts demonstrate that the sole requirement in the prevention of caries is the prevention of the formation of gelatinous plaques upon spots favoring their retention.

"It has been shown by Williams that caries is a reasonably slow process; therefore, the removal of plaques at frequent intervals is sufficient for the prevention of caries."

It is doubtful whether Williams or Black would go as far as this statement presumes to carry them. The editor might profitably have quoted Miller, who has demonstrated very conclusively that the so-called gelatinous plaques have little or nothing to do with the production of caries.

On results of secondary dentine the editor asserts that this tissue unquestionably brings about a degeneration in the pulp "which may become a cause of neuralgia." This statement requires further confirmation. It has been the observation of the reviewer that secondary dentine rarely or never results in neuralgia or other pathological conditions.

The editor still adheres to the use of aconite and iodine painted upon the gum in pericementitis. Aconite, as thus used, is of more than doubtful value, as its effect is to paralyze the sensory nerves of the part to which it is applied, and therefore produces results exactly the reverse of what is desired in a counterirritant.

The editor separates "interstitial gingivitis," as named by Talbot, from pyorrhœa alveolaris, making it a distinct disease. This is not as Talbot intended, for he distinctly renamed pyorrhœa alveolaris by this term, and it is difficult to understand how any pathologic distinctions can be made between these two as described by the editor.

In the treatment of this disease aromatic sulphuric acid is recommended. This is a very poor substitute for the commercial.

Leucoplakia is not mentioned in the book. This is a very important disease for dentists to become familiar with and be able to treat, or, at least, to advise a patient thus afflicted. This neglect of an important topic is to be regretted, as the dentist should be the first to call the patient's attention to this, as it is generally unnoticed. Intelligent advice in the earlier stages may save a surgical operation rendered necessary in severe cases.

The reviewer has felt it a duty to call attention to a few of the minor defects in this otherwise valuable production, but these do not militate against the book as a whole. It is regarded as covering the majority of the subjects included under the name of Dental Pathology with marked ability. It is, at present, the only work that can be recommended to dental students with a feeling that they can use it with profit to themselves as a text-book. It should be placed on the list of text-books in all dental colleges, and equally upon the shelves of the practitioner's library.

The book is sent out in the usual excellent style of Lea Brothers & Co.

Essentials of Materia Medica, Therapeutics, and Prescription Writing. By Henry Morris, M.D., Fellow of the College of Physicians of Philadelphia, etc. Sixth Edition, thoroughly revised by W. A. Bastedo, Ph.G., M.D., Tutor in Materia Medica in Columbia University. W. B. Saunders & Co., Philadelphia, New York, and London, 1904.

This small volume, while classed among the compends, richly deserves a higher place than is usually given works of this character. The original author of this book very clearly understood the value of a compend, for in the Preface to the first edition he says, "The Author hopes that, if properly used, this book will be of service to the student and young practitioner, but he is sure, from his experience as a teacher, that neither this or any other 'Compend' will suffice to form the groundwork of what is really the study of a lifetime." While this is true, a good compend is of great value to the student, probably more in this study than some others, for the acquisition of a knowledge of drugs, with the general student of medicine and dentistry, must largely depend on memory. The more thorough work must come later.

This book, in its sixth edition, covers most of the more recently introduced drugs; consequently it has a value for the dental operator, as well as to the student, as a convenient and reliable book of reference. For those who desire a more exhaustive treatment resort must be had to the larger works devoted to this subject.

